# Mechanical assessment of two hybrid plate designs for pancarpal canine arthrodesis under cyclic loading

**DOI:** 10.3389/fbioe.2023.1170977

**Published:** 2023-03-30

**Authors:** Ivan Zderic, Peter Varga, Ursula Styger, Ludmil Drenchev, Boyko Gueorguiev, Erik Asimus, W. Brian Saunders, Michael Kowaleski, Randy J. Boudrieau, Loic M. Déjardin

**Affiliations:** ^1^ AO Research Institute Davos, Davos, Switzerland; ^2^ Bulgarian Academy of Sciences, Institute of Metal Science, Equipment and Technologies with Center for Hydro- and Aerodynamics “Acad. A. Balevski”, Sofia, Bulgaria; ^3^ Ecole Nationale Vétérinaire de Toulouse, Toulouse, France; ^4^ College of Veterinary Medicine & Biomedical Sciences, Texas A&M University, College Station, TX, United States; ^5^ Cummings School of Veterinary Medicine, Tufts University, North Grafton, MA, United States; ^6^ College of Veterinary Medicine, Michigan State University, East Lansing, MI, United States

**Keywords:** pancarpal canine arthrodesis, hybrid plate, locking compression plate, radiocarpal hole, fatigue

## Abstract

Pancarpal canine arthrodesis (PCA) sets immobilization of all three carpal joints *via* dorsal plating to result in bony fusion. Whereas the first version of the plate uses a round hole (RH) for the radiocarpal (RC) screw region, its modification into an oval hole (OH) in a later version improves versatility in surgical application. The aim of this study was to mechanically investigate the fatigue life of the PCA plate types implementing these two features–PCA-RH and PCA-OH. Ten PCA-RH and 20 PCA-OH stainless steel (316LVM) plates were assigned to three study groups (n = 10). All plates were pre-bent at 20° and fixed to a canine forelimb model with simulated radius, RC bone and third metacarpal bone. The OH plates were fixed with an RC screw inserted either most proximal (OH-P) or most distal (OH-D). All specimens were cyclically tested at 8 Hz under 320 N loading until failure. Fatigue life outcome measures were cycles to failure and failure mode. Cycles to failure were higher for RH plate fixation (695,264 ± 344,023) *versus* both OH-P (447,900 ± 176,208) and OH-D (391,822 ± 165,116) plate configurations, being significantly different between RH and OH-D, *p* = 0.03. No significant difference was detected between OH-P and OH-D configurations, *p* = 0.09. Despite potential surgical advantages, the shorter fatigue life of the PCA-OH plate design may mitigate its benefits compared to the plate design with a round radiocarpal screw hole. Moreover, the failure risk of plates with an oval hole is increased regardless from the screw position in this hole. Based on these findings, the PCA plate with the current oval radiocarpal screw hole configuration cannot be recommended for clinical use.

## 1 Introduction

Pancarpal canine arthrodesis (PCA) sets immobilization of all three carpal joints—The antebrachiocarpal or radiocarpal, the middle carpal or intercarpal, and the carpometacarpal—To result in bony fusion of their joint surfaces (Buote et al., 2009; Ernst, 2012). It is a common and well-established salvage surgical procedure indicated for a variety of carpal disorders including hyperextension injuries, severe fractures, end-stage osteoarthritis, and neurologic deficits (Parker et al., 1981; Gambardella and Griffiths, 1982; Lesser, 2003), and is considered a standard of care procedure in small animal veterinary medicine with the goal to restore reasonable limb function.

Among different treatment options, such as pinning or external fixation, the most common PCA procedure relies on dorsal plating (Chambers and Bjorling, 1982). In this setting, the dorsally applied plate spans all carpal joints while being fixed with screws to the radius, the radiocarpal bone and usually the third metacarpal bone. Although a dorsally positioned plate lies on the compression side of the joints and is not favorable from a biomechanical perspective, dorsal plating is commonly performed due to the ease of the dorsal surgical approach compared to a palmar approach.

Carpal fusion angles between 0° (straight alignment) and 20° of extension have been proposed in the literature (Chambers and Bjorling, 1982; Guillou et al., 2012). The straight fusion alignment limits the risk of plate failure under dynamic loading, however, it is associated with both poorer paw placement during stance and increased tendon pain (Hottinger et al., 1996). Therefore, for a more physiological stance, a radiocarpal (RC) joint fusion angle of approximately 15° to 20° is clinically preferred, which requires bending of the PCA plate. This makes the bent plate more prone to fatigue failure (Lesser, 2003). Indeed, failure of these pancarpal arthrodesis plate types has been reported in literature with an incidence of 2%–9% (DeCamp et al., 1993; Bristow et al., 2015). Besides plate breakage, construct failures following dorsal plating occur due to screw breakage or loosening and/or bone fracturing at the peripheral ends of the plates (Johnson, 1980; Denny and Barr, 1991; Li et al., 1999; Whitelock et al., 1999). Furthermore, a mismatch between screw and metacarpal bone size is associated with increased risk of metacarpal bone fracture through the corresponding screw (Whitelock et al., 1999; Wininger et al., 2007) when dynamic compression plates (DCPs) or limited-contact DCPs (LC-DCPs) are used. These plates are also considered as too bulky for the distal metacarpal region, leading to increased wound dehiscence rates postoperatively (Worth and Bruce, 2008; Guillou et al., 2012).

**FIGURE 1:**
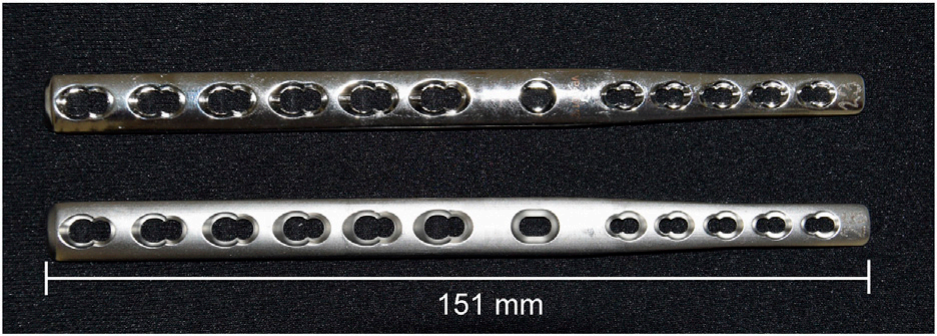
LCP Pancarpal Arthrodesis Plate 2.7/3.5 mm with round (up) and oval (down) standard radiocarpal screw hole, 12 holes, length 151 mm.

To overcome the limitations of compression plates, PCA plate designs were specifically devised (Guillou et al., 2012; Zderic et al., 2021). Among them, hybrid plates with distally tapered profile for both width and thickness became popular and accept application of smaller 3rd metacarpal screws for optimized stability and reduced risk of metacarpal fractures and wound dehiscence. However, screw loosening persisted as a major problem with standard compression plates not providing any mechanism to counteract this adverse effect (Clarke et al., 2009; Bokemeyer et al., 2011; Ernst, 2012). For this purpose, taking advantage of both the tapered design and locking plate technology, two new PCA plates were designed (Asimus et al., 2017; LCP Pancarpal Arthrodesis Plates - Surgical Technique, 2019). The locking plate design includes all combination plate holes to accommodate either standard or locking screws—Except the standard RC plate hole. The rationale for the latter is that this critical anchor point allows to angle the screw and ensure appropriate placement within the radiocarpal bone; Additionally, tightening of this screw results in pull-out of the RC bone towards the plate, preventing a caudal displacement of the RC bone. In one plate version, the non-locking RC plate hole was designed as a round hole (RH), while the other version features an oval “sliding” RC plate hole (OH) to allow for adjustments of the plate position (Figure 1), facilitate the surgical use of the plate, and provide improved placement of a screw into the RC bone. Although the mechanical superiority of the round-hole plate *versus* oval-hole plate was recently reported in a biomechanical study demonstrating increased plate surface strains next to the OH compared to the RH (Zderic et al., 2021), the biomechanical performance of both plate designs has not been investigated under cyclic loading, which warranted further evaluations under fatigue testing.

Therefore, the aim of this study was to investigate the mechanical behavior of the hybrid PCA plates with oval *versus* standard round RC plate hole under cyclic loading. We hypothesized that 1) Ovalization of the RC hole will decrease the fatigue life compared to the round-hole plate, and 2) Within the oval-hole plates, moving the screws from proximal to distal will further decrease the fatigue life and increase plate failure probability.

## 2 Methods

### Specimens and preparation

**FIGURE 2:**
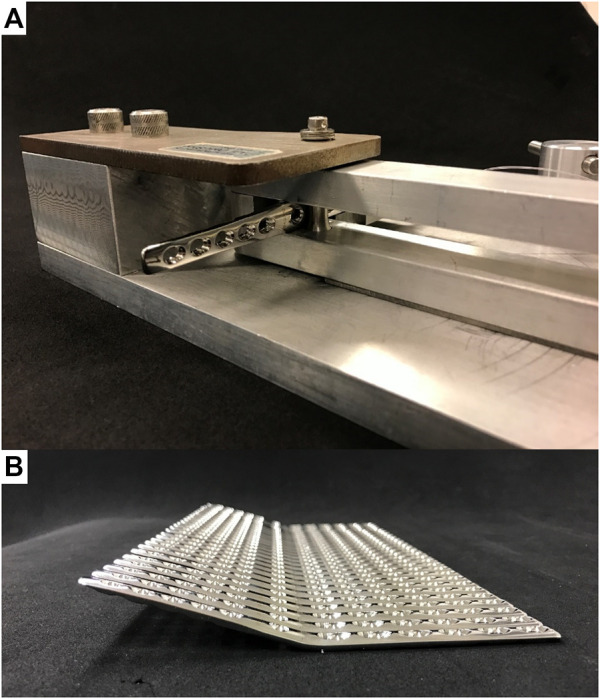
Pre-bending of round-hole PCA plates to 20° joint fusion angle; **(A)** Photograph showing a plate fixed within the bending press; **(B)** A consistent bending angle of all plates was obtained.

Ten RH and 20 OH hybrid PCA plates were consistently pre-bent to 20° joint fusion angle using a custom-made bending press (Figure 2). The press was designed to bend the plates consistently and accurately at a point centrally located between the distal radial and the RC holes, and was adapted from previous studies (Guillou et al., 2012; Zderic et al., 2021). All plates were fixed to three cylindric cotton fabric bone model substitutes (HGW 2082 [Canevasite], Amsler & Frey AG, Schinznach-Dorf, Switzerland) simulating the radius, RC bone and 3rd metacarpus with lengths resembling a cadaveric specimen (171, 10, and 123 cm, respectively) (Zderic et al., 2021). Each plate was fixed first to the radius by placing 3.5 mm locking screws in plate holes 1 (proximal), 3, 5, and 6, whereas the metacarpus was fixed second by placing 2.7 mm locking screws occupying holes 1 (proximal), 2, and 5. The RC bone was fixed last to the plate by occupying the RC screw hole with a standard 3.5 mm cortical screw. Ten OH plates were instrumented with the RC screw placed in the oval hole most proximally (OH-P) or most distally (OH-D). Pilot holes with diameters of 2.8, 2.0, and 2.5 mm were predrilled into the bone models prior to screw insertion. All 3.5 mm screws (locking and compression/cortex ones) were tightened at a final toque of 1.5 Nm, whereas the 2.7 mm locking screws were locked at 0.8 Nm. All implants were made of implant-grade stainless steel (316LVM), featuring a modulus of elasticity of 186 GPa, a yield strength of ≥690 MPa, and an ultimate tensile strength of 860–1100 MPa (Disegi and Eschbach, 2000), and were produced by the same manufacturer (DePuy Synthes, Zuchwil, Switzerland).

### Mechanical testing

**FIGURE 3:**
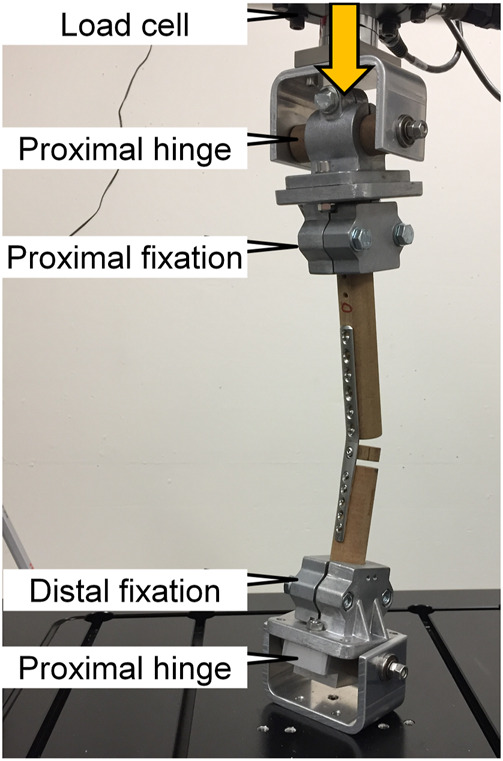
Test setup with a specimen mounted for mechanical testing, adopted from (Zderic et al., 2021).

Mechanical testing of the specimens was performed using an electrodynamic material testing machine (Acumen III, MTS Systems Corp., Eden Prairie, MN, United States) equipped with a 3 kN load cell. The test setup was adopted from a previous study and is shown in Figure 3 (Zderic et al., 2021). Articulated fixtures with co-axial alignment were used proximally and distally allowing free rotation in the sagittal plane. The distances between the bending point and the proximal and distal articulations of the fixtures were identical for all specimens. This guaranteed that the length of the proximal and distal lever arms with respect to the radio-carpal bone was consistent between specimens. Using a sine profile, the specimens were cyclically loaded in axial compression along the machine axis between 20 N valley load and 320 N peak compression at 8 Hz until construct failure. The latter was defined when the machine transducer reached a test stop criterion of 20 mm axial displacement with respect to its position at the beginning of the cyclic test. The force level and loading protocol were defined in a pilot study, where they were found appropriate to produce fatigue failures in RH plate constructs within a range between 500,000 and 1,000,000 cycles.

### Data acquisition and analysis

Strain levels inherent in the constructs under the given load magnitudes were assessed based on strain gauge data from a previously performed study (Zderic et al., 2021) measuring maximum plate surface strains on the same constructs under quasi-static loading to 300 N. Those results were considered to define the corresponding minimum strains at the valley load of 20 N, as well as to extrapolate them to the applied peak load level of 320 N. The minimum/maximum strains were further converted to the theoretical stress magnitudes, given the elastic modulus of 186 GPa for implant-grade stainless steel (316LVM) (Disegi and Eschbach, 2000). Furthermore, the strain-force curve of the previous study was considered to calculate the strain energy density of each specimen, taking into account the converted stresses. Finally, the maximum stress was normalized to the common mechanical property parameters for stainless steel, namely yield strength (690 MPa), ultimate tensile strength (1100 MPa), and fatigue strength (480 MPa) (Disegi and Eschbach, 2000).

Axial displacement and load were continuously recorded from the machine transducers at 64 Hz throughout testing. The numbers of cycles until construct failure were determined based on the recorded machine data. In addition, the mode of failure of each separate construct was recorded and subsequently analyzed.

Statistical analysis among the parameters of interest was performed using SPSS software package (version 27, IBM SPSS, Armonk, NY, United States). Mean and standard deviation (SD) were calculated for cycles to failure. Shapiro-Wilk test was conducted to ascertain a normal data distribution within all groups. One-Way Analysis of Variance (ANOVA) with Bonferroni *post hoc* test was conducted to identify significant differences between the groups. Pearson Correlation test was used to investigate the relation between maximum plate stresses and strain energy density. Level of significance set at *p* = 0.05.

## 3 Results

Outcome measures for maximum and minimum plate strains, together with the converted stresses, strain energy density, as well as maximum stress normalization to the common material mechanical parameters, are summarized in Table 1. For both OH configurations, peak plate stresses were overall the highest next to the RC screw and the non-occupied portion of the oval hole. Considering pooled data of all three groups together, the correlation between maximum strains and strain energy density was significant for each measured location (r ≥ 0.969, all *p* < 0.001). Maximum stresses remained below the yield and ultimate tensile strength in each group. However, the fatigue strength was exceeded for both OH configurations next to the RC screw and next to the non-occupied portion of the oval hole.

**TABLE 1:** Outcome measures based on previous experimental strain gauge measurements (Zderic et al., 2021), presented for each group separately in terms of mean value and SD.

Parameter	Plate Location	Group		
		RH	OH-D	OH-P
Maxiumum Strain at 320 N (µm/m)	Bending Point	2302.2 (121.8)	1926.0 (206.5)	1911.4 (185.7)
	Next to RC Screw	2264.6 (164.7)	3436.5 (497.8)	3551.1 (472.7)
	Slot	–	3588.6 (458.5)	3404.3 (461.2)
Maximum Stress at 320 N (MPa)	Bending Point	428.2 (22.7)	358.2 (38.4)	355.5 (34.5)
	Next to RC Screw	421.2 (30.6)	639.2 (92.6)	660.5 (87.9)
	Slot	–	667.5 (85.3)	633.2 (85.8)
Minimum Strain at 20 N (µm/m)	Bending Point	160.0 (40.9)	81.2 (39.0)	69.1 (13.6)
	Next to RC Screw	217.4 (40.0)	416.3 (208.3)	424.9 (194.5)
	Slot	–	358.7 (257.1)	286.6 (159.7)
Minimum Stress at 20 N (MPa)	Bending Point	29.7 (7.6)	15.1 (7.2)	12.9 (2.5)
	Next to RC Screw	40.4 (7.4)	77.4 (38.8)	79.0 (36.2)
	Slot	–	66.7 (47.8)	53.3 (29.7)
Strain Energy Density (J/m^3^)	Bending Point	987.1 (91.3)	721.2 (156.6)	714.0 (147.3)
	Next to RC Screw	934.0 (123.3)	2135.4 (577.1)	2268.6 (542.4)
	Slot	–	2369.5 (531.4)	2178.3 (549.8)
Maximum Stress Normalized to Yield Strength 690 MPa (%)	Bending Point	62.1 (3.3)	51.9 (5.6)	51.2 (5.0)
	Next to RC Screw	61.0 (4.45)	92.6 (13.4)	95.7 (12.7)
	Slot	–	96.7 (12.4)	91.8 (12.4)
Maximum Stress Normalized to Ultimate Tensile Strength 1100 MPa (%)	Bending Point	38.9 (2.1)	32.6 (3.5)	32.3 (3.1)
	Next to RC Screw	38.3 (2.8)	58.1 (8.4)	60.0 (8.0)
	Slot	–	60.7 (7.8)	57.6 (7.8)
Maximum Stress Normalized to Fatigue Strength 480 MPa (%)	Bending Point	89.2 (4.7)	74.6 (8.0)	74.1 (7.2)
	Next to RC Screw	87.8 (6.4)	133.2 (19.3)	137.6 (18.3)
	Slot	–	139.1 (17.8)	131.9 (17.9)

**FIGURE 4:**
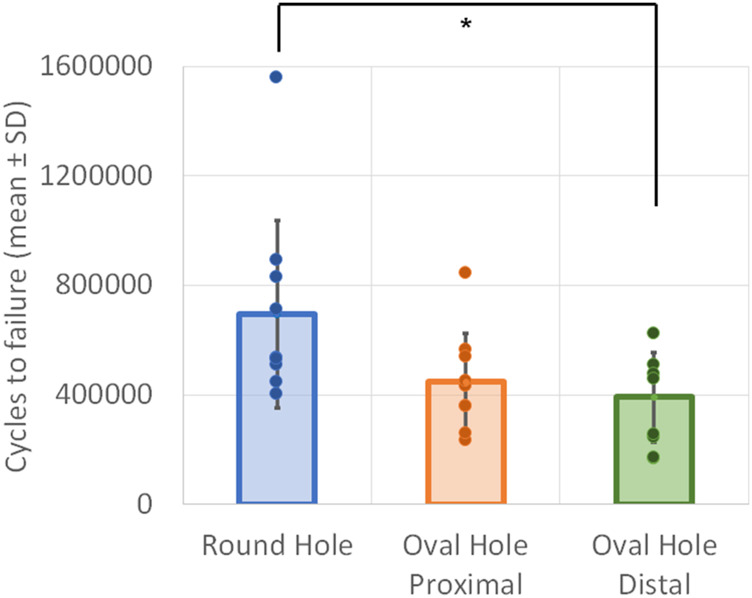
Cycles to failure in the three study groups presented in terms of mean value and SD. Circular points indicate cycles to failure for individual specimens. Star shows significant difference.

The RH plates exhibited the highest number of cycles to failure (695,264 ± 344,023 (mean ± SD), followed by OH-P (447,900 ± 176,208) and OH-D plates (375,954 ± 166,848). The values for RH plates were significantly higher *versus* OH-D plates (*p* = 0.028). No significant differences were detected between the OH-P and OH-D groups, *p* ≥ 0.999 (Figure 4). There was no significant difference in cycles to failure between the RH and OH-P plates (*p* = 0.092).

Constructs predominantly failed by plate fracture at the level of the distal radial combination hole in all groups (RH 6/10, OH-P 8/10, RH-D 8/10). In most cases the fracture occurred at the locking portion of the combination hole, i.e., the distal radial locking screw (RH 6/6, OH-P 5/8, RH-D 4/8), followed by fractures through the cortex screw portion of the distal radial combination hole (RH 0/6, OH-P 3/8, RH-D 3/8). In one OH-D specimen, the plate fractured through the RC screw hole. All plate fractures were initiated at the undersurface of the plate. Most plate fractures were located at a single locale on one flank next to a screw hole, whereas any fractures with two locales were on both flanks next to the same screw hole, but asymmetric, with one side exhibiting a larger fracture surface area. There were no plate fractures through the bending point of the plates. Plate fractures were predominantly associated with concurrent distal radial screw fractures at the head-shaft interface (RH 4/6, OH-P 4/8, RH-D 5/8).

The second most common failure mode was fracturing of the 1st and 2nd metacarpal screws at their head-shaft interface, associated with plate lifting-off from the bone model (RH 3/10, OH-P 2/10, RH-D 0/10), which occasionally ended in plate’s excessive bending at the 3rd metacarpal screw hole (RH 1/3, OH-P 1/2).

**FIGURE 5:**
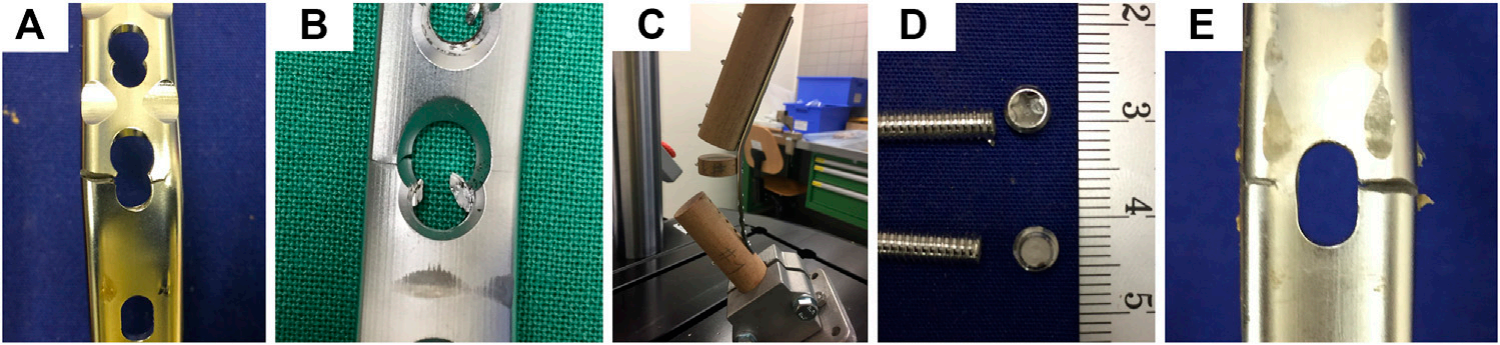
Photographs visualizing failure modes after fatigue testing; **(A)** asymmetric plate fracture through the locking part of a distal radial combination hole, posterior view; **(B)** asymmetric plate fracture through the compression part of a distal radial combination hole, anterior view. Debris in the locking part indicate fractured screw; **(C)** excessive plate bending at the most distal screw hole following 1st and 2nd metacarpal screw breakage, lateral view; **(D)** breakage of 3rd and 4th (distal) radial screws at their head-shaft interface, with numbers indicating length scale in centimeters; **(E)** asymmetric plate fracture through the radiocarpal hole, posterior view.

Finally, the third failure mode was a concomitant fracture of the 3rd and 4th (distal) radial screws at their head-shaft interface, which was associated with plate lifting-off from the radius bone model. Examples of plate failure modes are shown in Figure 5.

## 4 Discussion

This study evaluated the biomechanical performance of two hybrid locking plate designs for pancarpal canine arthrodesis with either a round or an oval RC hole design under cyclic loading. In addition, the effect of occupying the two extreme ends of the oval RC plate hole by a screw was investigated. The RH plates were associated with a higher fatigue life compared to both OH constructs, with a significant difference demonstrated between RH plates and OH-D plates. These results clearly demonstrate that the specific advantage of surgical maneuverability provided by the current iteration of PCA plates with an oval hole comes at the expense of reduced fatigue life. Bearing in mind the relatively high rates of reported implant related PCA failures (Johnson, 1980; Denny and Barr, 1991; Li et al., 1999), these findings should be considered when applying OH hole plates to clinical patients.

**FIGURE 6:**
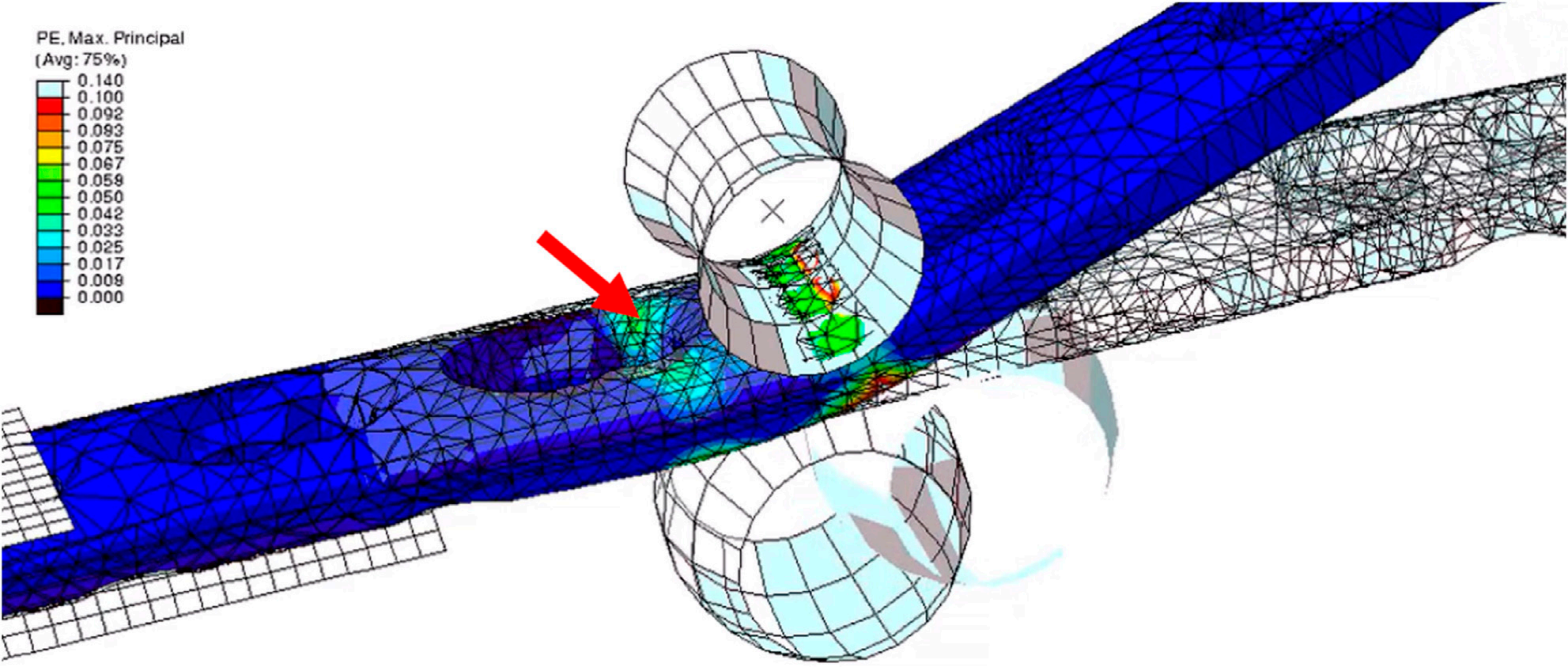
Finite element analysis of the plate pre-bending process demonstrating residual stresses after its completion (Zderic et al., 2021). Residual stresses also exist around the distal radial locking screw hole, as indicated by the red arrow, and are not limited to the bending point.

The reduced biomechanical performance characterizing OH plates is a logical consequence of the lowered area moment of inertia of the plate around its RC hole. However, it is counterintuitive that all but one failure modes occurred in the near periphery of the RC region, namely the distal radial combination hole or the proximal metacarpal holes. This is even more remarkable when considering the residual strains originating from plate contouring, amounting up to approximately 30%, as indicated by the finite element analysis from a previous study (Zderic et al., 2021), which would presume material weakening in this region (Figure 6). However, as demonstrated by this previous study, the residual stresses are not limited to the bending point but are also existent in proximity of the locking part of the distal radial combination hole. Indeed, these residual stresses may have contributed to the plate failure in this specific region. Other factors favoring these failure locations may be the used test setup, which did not permit contact between the three bone segments (Figure 3). The plate was not constrained in both the RC bone and bending point regions. Thus, using this test setup, the arthrodesis construct acts as a bridge plating construct with the working length defined by the distance of the innermost radial and metacarpal locking screws, upon which the transmitted forces are concentrated. In addition, the plates were pre-contoured to match the clinical process of plate application; however, pre-contoured plates are prone to axial bending leading to cumulated stresses on the affected screws along their nominal axes. These details may explain why plate lifting-off was a common failure mode. Interestingly, plate lift-off due to screw failure is a clinically observed mode of plate failure.

Although the failure modes were predominantly consistent within and across the groups, they were not unanimously reliable. This phenomenon can be ascribed to the many factors affecting the failure behavior. The constructs with pre-contoured plates were instrumented with multiple screws and subjected to complex axial loading. It is evident that the outcomes in terms of failure modes would disperse more compared to standardized tests using test coupons, although the parameters for specimen assembly were kept as reproducible as possible. The low number of failure modes detected in our study is attributed to similar failure loads, and each of them deems clinically relevant. In this regard, material contamination could be excluded as source of error, as evidenced by the failure analysis in a previous study, demonstrating that the material fulfilled the rigorous norms for implant-grade stainless steel (Zderic et al., 2022). As a result of these findings, the authors concluded that clinically translated plate failures can instead rather be ascribed to bone healing disturbances, leading to their extended loading and ultimate failure.

The relatively high fusion angle of 20° was certainly a contributing factor to plate lift-off. However, there is clinical evidence in the literature supporting a high fusion angle of 20°. A previous retrospective study compared the clinical outcome of pancarpal canine arthrodesis performed with two types of dorsally applied hybrid non-locking plates (Bristow et al., 2015). While the overall postoperative complication rates were similar for both plates (46%), implant failure was reported in 2% of the cases. However, the authors did not assess plate bending angle and since both plates feature a built-in 5° distal dorsal taper, it is reasonable to assume that the plates were applied with a 5° bend and without additional intra-operative bending. This assumption is supported by a high rate of postoperative lameness (up to 67%), regardless of plate type.

The locking screw mechanism represents another important factor for the failure behavior of the constructs. Given the angle-stable feature of this fixation, stresses are concentrated in the locking region. As a result, plate fractures through the locking portion of the combination hole with concomitant screw fracturing through its head-shaft interface would be expected as the most common failure mode.

The investigated plates are somewhat unique hybrid plates, first due to their tapered profile using different screw sizes proximally and distally, but also because of their ability to accommodate either locking or cortex screws, the latter being the only option in the RC hole. One potential solution to address the findings of the present study would be to modify the design of the RC hole with a variable-angle locking hole, allowing for angulated screw placement.

Considering the numbers of cycles to failure registered in this study, it must be acknowledged that fatigue-like behavior rather than true fatigue behavior was investigated. In this regard, no typical Wöhler curves could be generated based on different stress-strain tests, nor was it possible to indicate a stress level inherent in the plate cross-section due to the complex loading scenario and the inhomogeneous material cross-section. However, the load level was selected in a pilot study to achieve failure cycles according to recommended industry standards. True fatigue testing for steel would require cyclic loading over 1,000,000 cycles to reach its endurance limit (ASTM, 1999), although high-cycle fatigue tests are carried out for 10^7^ or more cycles (Campbell, 2012). In our study, we did not reach the endurance limit of the plates, which also was not targeted, as an aggressive loading protocol, tailored to mimic physiological loading, is required to estimate and compare the mechanical competence of the tested constructs. Arthrodesis is achieved on average within 12 postoperative weeks, but can range from 9 to 30 weeks (Michal et al., 2003). The corresponding number of load cycles lies within a relatively broad range between approximately 200,000 cycles and 1,600,000 cycles. This would correspond to a period of approximately 12–96 weeks, if activity is extrapolated from humans (ASTM, 1999). An overlapping of these two ranges indicates that a clinical failure cannot be excluded. These presumptions should be considered as worst-case scenarios, as the calculations were rather conservative, neglecting the gradual increase in load bearing of the bone healing over time.

The applied peak force of 320 N was selected based on front limb ground reaction force of approximately 115% body weight. Considering that the plates used in this study would be appropriate for dogs weighing between 16 kg and 46 kg, a 320 N peak load would simulate loading conditions for an approximately 28 kg mid-size dog (Li et al., 1999; Clarke et al., 2009; Andreoni et al., 2010).

The present study built upon a previous mechanical evaluation of PCA plates (Zderic et al., 2021). Indeed, plates were instrumented with strain gauges at the most sensitive locations, as predicted by a previous finite element analysis. Constructs were then loaded in the same fashion under axial quasi-static compression over 10 cycles. Although a validated finite element model accurately predicted the weakened properties associated with the oval plate hole, actual failure locations were not precisely identified. This probably occurred because the authors’ focus was limited to the RC region, neglecting the peripheral boundary conditions at the distal radial and proximal metacarpal screw holes.

The peak principal stresses, as theoretically converted from experimentally measured peak plate strains, remained unexceptionally below the yield strength, and predominantly below the fatigue strength. These findings highlight the benefits of strain gauges measurements, which remain indispensable tools for prediction of failure loads and determination of correct load magnitudes in studies investigating the fatigue behavior of different constructs.

The present and the abovementioned studies represent the only studies on arthrodesis plates using locking technology. Locking compression plates were initially designed to increase construct stability in poor bone quality and reduce the rates of screw loosening (Auer et al., 1995). In good bone quality, such as in the canine forelimb simulated here, the potentially overly stiff configuration may lead to different effects than expected, namely to earlier construct failure as also suggested in previous studies (Fitzpatrick et al., 2009; Rowe-Guthrie et al., 2015).

The test setup in the current study was similar to that used in a previous biomechanical comparison of non-locking hybrid plates *versus* LC-DCPs (Guillou et al., 2012). Other biomechanical studies focused on the mechanical characterization of locking and non-locking plates (Wininger et al., 2007; Meeson et al., 2012; Rothstock et al., 2012; Rowe-Guthrie et al., 2015). In a recent study, Meeson et al. (2012) subjected plates to quasi-static load to failure and fatigue loading over 10^6^ cycles in two independent test series of four-point bending, concluding that fatigue failure during the convalescence period of estimated 150,000–200,000 cycles is unlikely. However, the authors only considered straight plates and the consequences of pre-contouring was not investigated. To date, studies investigating the effect of residual stresses emerging from plate pre-contouring on their fatigue performance are lacking. We therefore anticipate that this investigation, by means of dedicated destructive, semi-destructive or non-destructive techniques, may unveil new insights into this insufficiently explored area.

As with all studies, this study is not without limitations. First, an artificial bone model was used with material properties, which may not represent the complex material characteristics of several canine bones. This simplification however helped to determine the plates’ fatigue properties. Second, the bone configuration was chosen to simulate a worst-case scenario with no bony interaction between the radiocarpal bone and the radius or metacarpals, which in most clinical cases does not represent the position or contribution of the RC bone relative to the remaining stability of the antebrachial bones. Nonetheless, this model mimics the immediate and short-term postoperative periods prior to bone healing contributing to construct stability. Third, streamlined uniaxial loading was applied at constant load magnitude, whereas the complex multidirectional forces occurring during a true gait were neglected, primarily since these forces in the canine antebrachium remain largely unknown. Fourth, there was no clear association between the number of cycles to failure and the corresponding mode of failure. Again, this reflects to some extent the unpredictable nature of cyclic testing.

Future studies should focus on three aspects to broaden the knowledge of locking arthrodesis plates. First, a direct comparison to non-locking plates is missing and conclusions on the effectiveness of the locking principle cannot be drawn. Of paramount importance would therefore be to provide evidence of their effectiveness over compression plating in terms of implant loosening. Second, the effect of plate pre-shaping on the fatigue behavior remains to be explored and could help guide surgeons regarding the degree of acceptable plate pre-contouring. This could be accomplished by comparing the structural and mechanical properties of plates pre-contoured at different angles. Third, plate design iterations with reinforced flanks around the oval RC hole may enhance the fatigue life while preserving the positive features of the oval sliding hole.

## 5 Conclusion

From a biomechanical perspective, despite the surgical advantages of the PCA plate containing an oval radiocarpal screw hole, its fatigue life under the pre-defined load magnitude of 320 N is shorter when compared to the plate design with a round radiocarpal screw hole and therefore appears to mitigate the clinical benefit of the oval hole. Moreover, the failure probability of the oval RC hole plate is increased regardless of the position of the screw in this hole. Based upon these findings, the current iteration of the PCA plate with an oval radiocarpal screw should be used with caution, and additional design features are likely necessary to increase the fatigue life of oval RC-hole PCA plates.

## Data Availability

The raw data supporting the conclusion of this article will be made available by the authors, without undue reservation.
